# Multiple Energy Transfer Channels in Rare Earth Doped Multi‐Exciton Emissive Perovskites

**DOI:** 10.1002/advs.202307354

**Published:** 2023-12-21

**Authors:** Huwei Li, Kai Han, Zheyu Li, Hongxia Yue, Xinyu Fu, Xinyu Wang, Zhiguo Xia, Shuyan Song, Jing Feng, Hongjie Zhang

**Affiliations:** ^1^ State Key Laboratory of Rare Earth Resource Utilization Changchun Institute of Applied Chemistry Chinese Academy of Sciences Changchun Jilin 130022 China; ^2^ State Key Laboratory of Inorganic Synthesis and Preparative Chemistry College of Chemistry Jilin University Changchun Jilin 130012 China; ^3^ State Key Laboratory of Luminescent Materials and Devices School of Physics and Optoelectronics South China University of Technology Guangzhou Guangdong 510641 China; ^4^ School of Applied Chemistry and Engineering University of Science and Technology of China Hefei Anhui 230026 China; ^5^ Department of Chemistry Tsinghua University Beijing 100084 China

**Keywords:** energy transfer, Förster–Dexter theory, halide perovskites, multi‐exciton, rare earth

## Abstract

Revealing the energy transfer (ET) process from excitons to rare earth ions in halide perovskites has great guiding value for designing optoelectronic materials. Here, the multiple ET channels in multi‐exciton emissive Sb^3+^/Nd^3+^ co‐doped Cs_2_ZrCl_6_ are explored to comprehend the ET processes. Förster–Dexter ET theory reveals that the sensitizer concentration rather than the overlap integral of the spectra plays the leading function in the comparison of the ET efficiency among multiple ET channels from the host self‐trapped excitons (STEs) and dopant triplet STEs to Nd^3+^ ions. Besides, Sb^3+^/Nd^3+^ co‐doped Cs_2_ZrCl_6_ enables varied color delivery and has great potential as anti‐counterfeiting material. Under X‐ray irradiation, Sb^3+^/Nd^3+^ co‐doped Cs_2_ZrCl_6_ presents a high light yield of ≈13300 photons MeV^−1^ and promising X‐ray imaging ability. This work provides new insight for investigating the ET efficiency among multiple ET processes and presents great potentiality of multi‐exciton emissive perovskites in the fields of anti‐counterfeiting and X‐ray imaging.

## Graphical abstract

**F**örster–Dexter energy transfer (ET) theory reveals that the sensitizer concentration rather than the overlap integral of the spectra plays the leading function in the comparison of the ET efficiency among multiple ET channels from the host self‐trapped excitons (STEs) and dopant triplet STEs to Nd^3+^ ions in Cs_2_ZrCl_6_:Sb^3+^,Nd^3+^. Besides, Cs_2_ZrCl_6_:Sb^3+^,Nd^3+^ presents great potentiality in the fields of anti‐counterfeiting and X‐ray imaging.

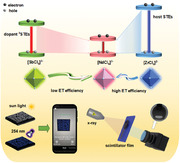

## 1 Introduction

Self‐trapped excitons (STEs) widely exist in alkali metal halide crystals, silica, and simple organic molecular crystals, and form in the excited‐state, where electron–phonon coupling is strong enough for excited electrons and holes to severely deform the lattice to create a potential well and trap the excitons in it.^[^ ^1^, ^2^, ^3^, ^4^, ^5^ ^]^ Particularly in past few years, STEs play a leading function in improving the optical property of lead‐free perovskites, which usually display low photoluminescence quantum yields (PLQYs) due to the indirect bandgap or the parity‐forbidden transition in direct bandgap perovskites.^[^ ^6^, ^7^, ^8^, ^9^, ^10^, ^11^, ^12^, ^13^ ^]^ STEs in lead‐free perovskites could be divided into intrinsic STEs and extrinsic STEs. The former exists in lead‐free perovskite host and the latter usually could be induced via introducing ns^2^ dopants, such as Sb^3+^, Bi^3+^, Te^4+^, etc.^[^ ^14^, ^15^, ^16^, ^17^, ^18^, ^19^, ^20^, ^21^, ^22^ ^]^

Apart from improving the luminescent performance of lead‐free perovskites, STEs could sensitize other emission centers (rare earth ions, RE^3+^; manganese ions, Mn^2+^, etc.) via the energy transfer (ET) channel.^[^ ^23^, ^24^, ^25^, ^26^, ^27^, ^28^ ^]^ A part of these luminescent ions, such as Sm^3+^, Er^3+^, Tm^3+^, Nd^3+^, and Ho^3+^, always exhibit weak light absorption caused by the electric‐dipole‐forbidden of 4f → 4f transitions, which limits their downshifting luminescent performance. However, building the ET channels from the STEs to these ions could overcome this drawback because the STEs always possess broad and intense UV or blue light absorption. As a consequence, the ET channel has been studied deeply and employed to tune the optical performance of lead‐free perovskites in the visible and near infrared region (NIR).^[^ ^29^, ^30^, ^31^ ^]^ Meanwhile, previous reports focused on the ET channel from single STEs to single or multiple emission centers. As far as we know, there is a lack of sufficient research on the ET channels from multiple STEs to single emission center and the efficiency of different ET processes in rare earth doped multi‐exciton emissive perovskites. It is challenging and important to explore and understand these multiple ET processes for designing optoelectronic materials.

Compared with those common lead‐free perovskites with two dopant extrinsic STEs, such as Cs_2_SnCl_6_:Sb^3+^ and Cs_2_NaInCl_6_:Sb^3+^/Bi^3+^, 0D lead‐free perovskite Cs_2_ZrCl_6_:Sb^3+^ with a host intrinsic STEs and two dopant extrinsic STEs was selected as multi‐exciton emissive material for the more abundant STEs.^[^ ^32^, ^33^, ^34^, ^35^ ^]^ According to the previous reports, the three kinds of STEs in Cs_2_ZrCl_6_:Sb^3+^ could be assigned to the host intrinsic STEs in the [ZrCl_6_]^2−^ octahedra, the singlet and triplet dopant extrinsic STEs in the [SbCl_6_]^3−^ octahedra, respectively. Among common emission centers (RE^3+^, Mn^2+^, etc.), Nd^3+^ ions as an intense NIR emitter could be sensitized by the STEs in perovskite and have no visible emission that would overlap and disturb the broad STEs emissions.^[^ ^36^ ^]^ Consequently, it is feasible for choosing Sb^3+^/Nd^3+^ co‐doped Cs_2_ZrCl_6_ perovskite to investigate the different ET processes between multiple STEs and other emission centers.

Herein, we fabricated Sb^3+^/Nd^3+^ co‐doped Cs_2_ZrCl_6_ microcrystals (MCs) with three STEs emissions and Nd^3+^ NIR emission through room temperature precipitation method. The efficiency of multiple ET processes from the host STEs and dopant STEs to Nd^3+^ ions was investigated by the time‐resolved photoluminescence (PL) decays. Förster–Dexter ET theory demonstrates the sensitizer concentration rather than overlap integral of the PL spectrum of sensitizer and the absorption spectrum of activator plays the dominant role in the comparison of the ET efficiency among multiple ET channels from the different STEs to Nd^3+^ ions. Furthermore, Sb^3+^/Nd^3+^ co‐doped Cs_2_ZrCl_6_ MCs display the great potential as anti‐counterfeiting material and X‐ray scintillator.

## 2 Results

**Figure 1:**
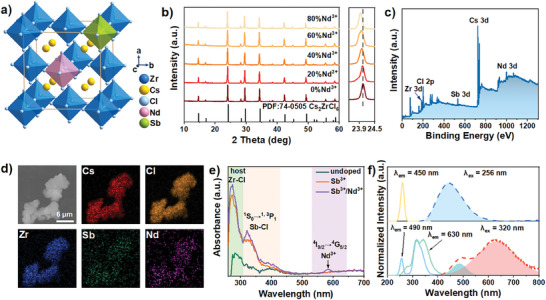
a) Schematic of the cubic crystal structure of Cs_2_ZrCl_6_:Sb^3+^,Nd^3+^ MCs. b) Powder XRD patterns (left panel) and magnified XRD peaks (right panel) of Cs_2_ZrCl_6_:0.5%Sb^3+^,Nd^3+^ MCs. c) XPS spectrum of Cs_2_ZrCl_6_:0.5%Sb^3+^,80%Nd^3+^. d) SEM image and elemental mappings of Cs_2_ZrCl_6_:0.5%Sb^3+^,80%Nd^3+^ MCs. e) UV–vis absorption spectra of undoped, Sb^3+^ doped, and Sb^3+^/Nd^3+^ co‐doped Cs_2_ZrCl_6_ MCs. f) Normalized PL (dashed lines) and PLE (solid lines) spectra of undoped (top) and 0.5% Sb^3+^ doped Cs_2_ZrCl_6_ MCs (bottom).

Sb^3+^/Nd^3+^ co‐doped Cs_2_ZrCl_6_ MCs were synthesized through a coprecipitation method in a mixed solution of hydrochloric acid and methanol at room temperature. The actual dopant concentrations in Sb^3+^ doped and Sb^3+^/Nd^3+^ co‐doped Cs_2_ZrCl_6_ MCs were ascertained by inductively coupled plasma optical emission spectrometry (ICP‐OES), which provided a much lower actual Nd^3+^ concentrations than its feeding concentrations due to the quite different solubility of RE halides from other metal halides in mixture solution (Tables S1 and S2, Supporting Information). Sb^3+^/Nd^3+^ co‐doped Cs_2_ZrCl_6_ MCs possess vacancy‐ordered double perovskite structure (space group *Fm* $\bar{3}$ *m*), in which isolated [ZrCl_6_]^2−^ octahedra are surrounded by Cs^+^ cations (**Figure** **1**a).

After doping Nd^3+^ and Sb^3+^ ions, the formed [SbCl_6_]^3−^ octahedra and [NdCl_6_]^3−^ octahedra can partially replace [ZrCl_6_]^2−^ octahedra. In Figure 1b and Figure S1 (Supporting Information), the powder X‐ray diffraction (XRD) patterns of Cs_2_ZrCl_6_:Sb^3+^ and Cs_2_ZrCl_6_:0.5%Sb^3+^,Nd^3+^ MCs agree well with the standard pattern of cubic Cs_2_ZrCl_6_ (PDF#74‐0505), indicating all the prepared MCs are pure phase without impurity. Meantime, with the increasing concentrations of Sb^3+^ or Nd^3+^ ions, the characteristic diffraction peak (220) shifts gradually to lower angle, implying the expansion of lattice due to the larger ion radius of Sb^3+^ ions (0.76 Å, CN = 6) and Nd^3+^ ions (0.98 Å, CN = 6) than that of Zr^4+^ ions (0.72 Å, CN = 6).^[^ ^37^ ^]^ X‐ray photoelectron spectroscopy (XPS) identifies the presence of Cs^+^, Zr^4+^, Cl^−^, Sb^3+^, and Nd^3+^ in Cs_2_ZrCl_6_:Sb^3+^,Nd^3+^ MCs, indicating the successful doping of Nd^3+^ and Sb^3+^ ions (Figure 1c). The SEM image and the elemental mappings reveals homogeneous distribution of Cs^+^, Zr^4+^, Cl^−^, Sb^3+^, and Nd^3+^ in Cs_2_ZrCl_6_:Sb^3+^,Nd^3+^ MCs (Figure 1d).

The optical properties of as‐prepared Zr‐based MCs were investigated. In Figure 1e, the absorption spectra reveal the additional overlapping double peaks ranging from 300 to 425 nm, corresponding to the ^1^S_0_ → ^1, 3^P_1_ transitions of Sb^3+^ ions. Besides, a narrow absorption peak at 584 nm only observed in Nd^3+^/Sb^3+^ co‐doped Cs_2_ZrCl_6_ MCs could be ascribed to ^4^I_9/2_ → ^4^G_5/2_ transition of Nd^3+^ ions.^[^ ^38^ ^]^ Then, the PL and PL excitation (PLE) spectra of undoped and Sb^3+^ doped Cs_2_ZrCl_6_ MCs were carried out (Figure 1f and Figure S2, Supporting Information). Two broad PL peaks at 490 and 630 nm in the PL spectra of Cs_2_ZrCl_6_:Sb^3+^ excited by 320 nm correspond to the singlet STEs (^1^STEs) and the triplet STEs (^3^STEs) in the [SbCl_6_]^3−^ octahedra, respectively. As the increasing of Sb^3+^ ions concentration, the two dopant STEs emissions become stronger and ^3^STEs emission become more dominant than ^1^STEs emission at the meantime, which may be ascribed to the enhanced ET process from ^1^STEs to ^3^STEs.^[^ ^35^ ^]^ Besides, under the excitation of 256 nm, the PL spectra of Cs_2_ZrCl_6_:Sb^3+^ exhibit the weak emission peaked at 450 nm that also exists in the PL spectrum of undoped MCs, corresponding to the host STEs emission in the [ZrCl_6_]^2−^ octahedra. The decreased PL intensity of host STEs emission in Cs_2_ZrCl_6_:Sb^3+^ as the increase of Sb^3+^ doping concentration could be ascribed to the increasing defect density caused by Sb^3+^ ions rather than the ET process from host STEs to dopant STEs, because the emission processes of them are mutually independent, which could be largely attributed to the hindered charge carriers transport between the neighboring octahedra in Cs_2_ZrCl_6_:Sb^3+^ perovskite.^[^ ^34^ ^]^ The PLE spectrum of undoped Cs_2_ZrCl_6_ monitored at 450 nm exhibit a single PLE peak at 256 nm that corresponds to the Zr ─Cl charge transfer transition in the [ZrCl_6_]^2−^ octahedra. Besides, for the emissions at 490 and 630 nm, the PLE spectra of Cs_2_ZrCl_6_:0.5%Sb^3+^ show the different patterns. The former exhibits two PLE peaks at 256 and 320 nm, corresponding to the Zr─Cl charge transfer transition and ^1^S_0_ → ^1^P_1_ transition of Sb^3+^ ions, respectively. Here, the existence of PLE peak at 256 nm in Cs_2_ZrCl_6_:0.5%Sb^3+^ could be ascribed to the distribution of host STEs at 490 nm. The latter consists of a doublet (peak fitting 1, 277 nm; peak fitting 3, 340 nm) band and a weaker band (peak fitting 2, 310 nm), corresponding to the ^1^S_0_ → ^3^P_1_ and ^1^S_0_ → ^1^P_1_ transitions of Sb^3+^ ions, respectively (Figure S2d, Supporting Information). Interestingly, the doublet band shows a greater splitting than that of Sb^3+^‐doped Cs_2_NaInCl_6_ in previous reports (≈20 nm for Sb^3+^‐doped Cs_2_NaInCl_6_; ≈63 nm for Sb^3+^‐doped Cs_2_ZrCl_6_), implying a dynamic Jahn–Teller distortion of the excited‐state (^3^P_1_) with a greater extent for crystal environment that is more prone to octahedral distortion.^[^ ^39^, ^40^, ^41^ ^]^ Moreover, the PLE peak attributed to ^1^S_0_ → ^1^P_1_ transition exists in the PLE spectrum of Cs_2_ZrCl_6_:0.5%Sb^3+^ monitored at 630 nm, implying the ET from the singlet excited‐state (^1^P_1_) to the triplet excited‐state (^3^P_1_).

**Figure 2:**
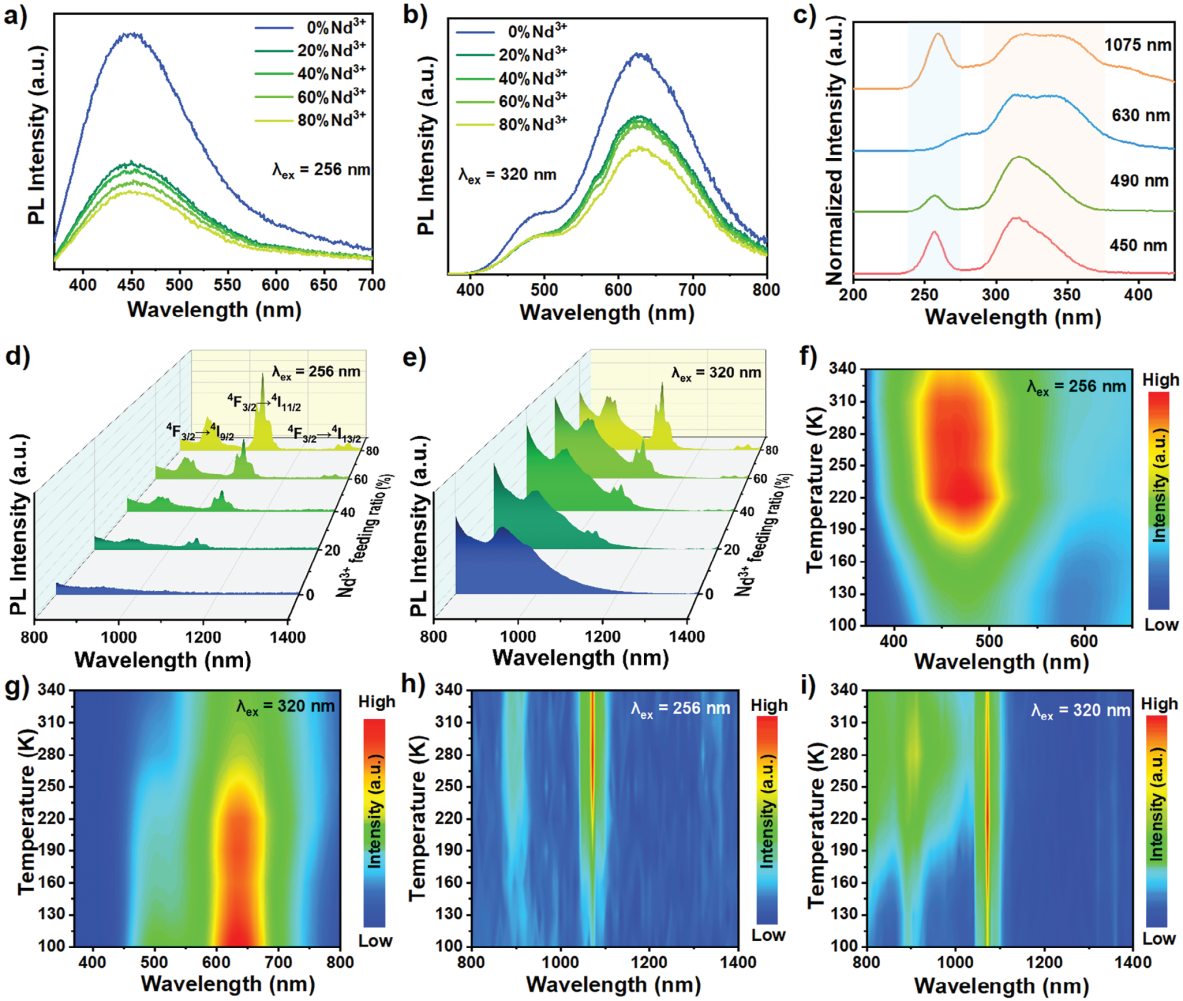
PL spectra of Cs_2_ZrCl_6_:0.5%Sb^3+^,(0–80%)Nd^3+^ MCs in the visible region excited by a) 256 nm and b) 320 nm. c) Normalized PLE spectra of Cs_2_ZrCl_6_:0.5%Sb^3+^,80% Nd^3+^ MCs monitored at different wavelength. PL spectra of Cs_2_ZrCl_6_:0.5%Sb^3+^,(0–80%)Nd^3+^ MCs in NIR region excited by d) 256 nm and e) 320 nm. PL spectra versus temperature of Cs_2_ZrCl_6_:0.5%Sb^3+^,80% Nd^3+^ MCs in visible region excited by f) 256 nm and g) 320 nm. PL spectra versus temperature of Cs_2_ZrCl_6_:0.5%Sb^3+^,80% Nd^3+^ MCs in NIR region excited by h) 256 nm and i) 320 nm.

After Nd^3+^ ions are introduced into Cs_2_ZrCl_6_:0.5%Sb^3+^ MCs, sharp NIR emission appears resulting from the ET from STEs to Nd^3+^ ions. The PL spectra of Cs_2_ZrCl_6_:0.5%Sb^3+^,(20–80%)Nd^3+^ MCs excited by 256 and 320 nm are shown in **Figure** **2**a,b,d,e. As the increase of Nd^3+^ concentration, both the host STEs and dopant ^3^STEs emissions decline to a great extent along with the rising of Nd^3+^ NIR emission, implying the possibility of ET processes from the host STEs and dopant ^3^STEs to Nd^3+^ ions. Interestingly, compared with the PL intensity the host STEs and ^3^STEs, that of ^1^STEs exhibits slight decline with the Nd^3+^ ions concentration increasing, implying that ^1^STEs may contribute negligible carriers to Nd^3+^ ions and indicating that the Nd^3+^ concentration could tune the relative intensity of ^1^STE and ^3^STE emissions (Figure S3, Supporting Information). The ET channel could be further investigated by the PLE spectra. In Figure 2c, the PLE spectrum of Cs_2_ZrCl_6_:0.5%Sb^3+^,80%Nd^3+^ monitored at 1075 nm exhibits a broad band (275–400 nm) and a narrow peak at 256 nm. The former shares similar excitation pattern with that of ^3^STEs emission measured at 630 nm while the latter originates from the Zr─Cl charge transfer transition and could also be observed in the PLE spectrum monitored at 450 nm, implying that Nd^3+^ share the same excited‐state with the host STEs and dopant ^3^STEs, and Nd^3+^ emission is sensitized by the ET from the host STEs and dopant ^3^STEs to Nd^3+^ ions rather than direct excitation of Nd^3+^ ions. Besides, compared with that monitored at 490 nm, the PLE spectrum monitored at 1075 nm does not show a prominent PLE peak at 320 nm (^1^S_0_ → ^1^P_1_), indicating the negligible ET contribution from ^1^STEs.

The PL spectra recorded at 100–340 K could also prove and understand the ET process in Cs_2_ZrCl_6_:0.5%Sb^3+^,80% Nd^3+^ MCs. Excited by 256 nm, the host STEs emission become stronger (100–220 K) and then weaker (220–340 K) with the increasing temperature (Figure 2f). This abnormal behavior may be attributed to the competition between thermal‐induced PL quenching and thermally activated delayed fluorescence, the latter involves the reverse intersystem crossing between the triplet and singlet excited‐states.^[^ ^42^, ^43^ ^]^ Meantime, under the excitation at 256 nm, the PL intensity of Nd^3+^ NIR emission first increases (100–280 K) and decreases subsequently (280–340 K) with the increasing temperature (Figure 2h). This phenomenon could be ascribed to the competition between thermal‐induced PL quenching and carrier transition to Nd^3+^ ions, implying the existence of ET process from the host STEs to Nd^3+^ ions.^[^ ^44^ ^]^ Besides, excited by 320 nm, the PL intensity of ^1^STEs emission gradually decreases with the increase of temperature from 100 to 340 K, however, the ^3^STEs emission undergoes an abnormal increase from 160 to 190 K, which could be ascribed to the ET from the excited‐state ^1^P_1_ to ^3^P_1_ upon increasing the temperature (Figure 2g; Figure S4, Supporting Information). In addition to this abnormal increase phenomenon, its PL intensity decreases gradually in the range of 100–160 K and 190–340 K. Furthermore, the Nd^3+^ emission excited by 320 nm first rises (from 100 to 220 K) and declines subsequently (from 220 to 340 K), and the similar phenomenon implies the ET channel from ^3^STEs to Nd^3+^ ions (Figure 2i). In combination with the results mentioned above, it could be demonstrated the existence of two ET channels from the host STEs and dopant ^3^STEs to Nd^3+^ ions.

**Figure 3:**
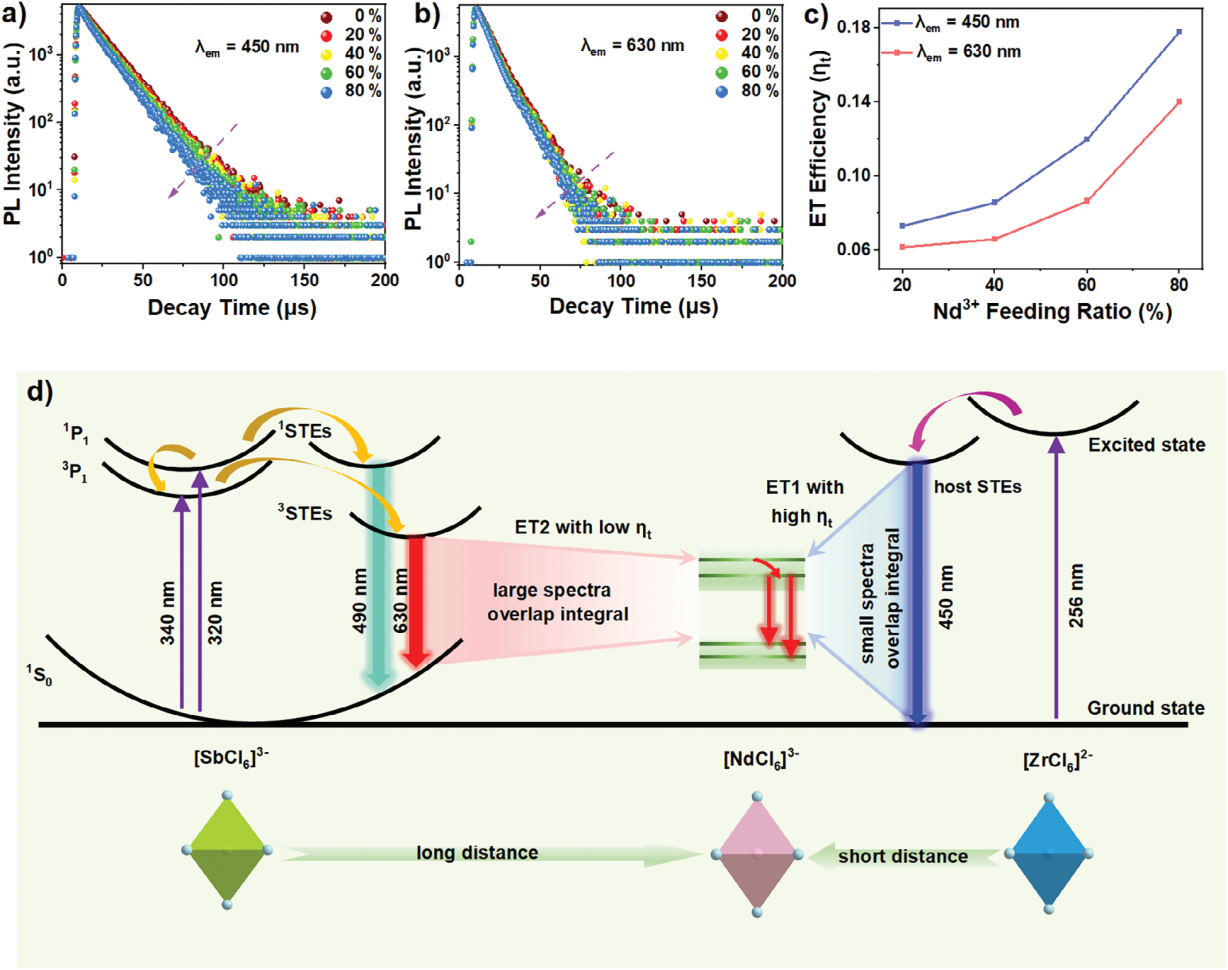
The decay curves of Cs_2_ZrCl_6_:0.5%Sb^3+^, (0–80%) Nd3^+^ MCs monitored at a) 450 nm and b) 630 nm. c) The ET efficiency (*η*_t_) variation of Cs_2_ZrCl_6_:0.5%Sb^3+^,(20–80%)Nd^3+^ MCs (monitored at 450 and 630 nm) as a function of Nd^3+^ feeding ratio. d) Schematic diagram of the proposed multiple ET model.

To further understand the ET process and investigate the ET efficiency, the time‐resolved PL decays of Cs_2_ZrCl_6_:0.5%Sb^3+^,(0–80%)Nd^3+^ MCs were carried out. As exhibited in **Figure** **3**a, after being fitted by the mono‐exponential function, the PL decay curves of Cs_2_ZrCl_6_:0.5%Sb^3+^,Nd^3+^ monitored at host STEs emission (λ_ex_ = 256 nm) could provide the lifetimes of 14.82, 13.74, 13.55, 13.05, and 12.19 µs, corresponding to the Nd^3+^ concentrations of 0%, 20%, 40%, 60%, and 80%, respectively. These lifetimes match well with the previous reports and could be attributed to the host STEs recombination.^[^ ^34^ ^]^ Similarly, as shown in Figure 3b and Table S3 (Supporting Information), the PL decay curves monitored at 630 nm provide a series of lifetimes after fitted by mono‐exponential function, which can be assigned to the dopant ^3^STEs recombination. It could be observed that with the increasing Nd^3+^ ions concentration, the lifetimes of both host STEs and dopant ^3^STEs decrease due to the ET from them to Nd^3+^ ions. To further compare these ET processes, the ET efficiency *η*_t_ was adopted and can be obtained by the following equation:

(1) $$\eta_{t}=1-\frac{\tau_{x}}{\tau_{s}}$$

where τ_x_ and τ_s_ are the lifetimes of STEs in the presence or absence of Nd^3+^ ions.^[^ ^25^ ^]^ As shown in Figure 3c, with Nd^3+^ concentration increasing, the ET efficiency *η*_t_ increases gradually and host STEs have the higher value of *η*_t_ than that of dopant ^3^STEs at all Nd^3+^ feeding concentrations (20%, 40%, 60%, and 80%).

Furthermore, according to Förster–Dexter theory, the ET probability *P*_SA_ can be estimated by the following expressions^[^ ^45^, ^46^ ^]^:

(2) $$P_{\mathrm{SA}}=\frac{1}{\tau_{s}}{\left(\frac{R_{0}}{r}\right)}^{6}$$

(3) $$P_{\mathrm{SA}}=\frac{1}{\tau_{s}}\left(\frac{\eta_{t}}{1-\eta_{t}}\right)$$

where *R*_0_ is the critical distance and *r* is the distance between sensitizer and activator. To our knowledge, the dopants are uniformly distributed in the lattice and the three cations (Zr^4+^, Sb^3+^, and Nd^3+^) share the same lattice sites in the unit cell, hence, the distance *r* between sensitizer and activator can be estimated according to Blasse's equation ^[^ ^47^ ^]^:

(4) $$r=2{\left(\frac{3V}{4\pi Nc}\right)}^{1/3}$$

where *c* is the total concentration of sensitizer and activator, *N* is the number of host cations that might be occupied by the activator and sensitizer in the unit cell, and *V* is the volume of the unit cell. Then, Equations (2) and (4) were substituted into Equation (3) to obtain Equation (5) as follows:

(5) $$\frac{\eta_{t}}{1-\eta_{t}}=\frac{\pi^{2}N^{2}R_{0}^{6}c^{2}}{36V^{2}}$$

Then, the related parameters of two ET processes in Cs_2_ZrCl_6_:0.5%Sb^3+^,Nd^3+^ were substituted into Equation (5) to obtain Equation (6).

(6) $$\left(\frac{\eta_{t1}}{1-\eta_{t1}}\right)/\left(\frac{\eta_{t2}}{1-\eta_{t2}}\right)=\frac{R_{01}^{6}}{R_{02}^{6}}\times {\left(\frac{c_{Nd}+c_{Zr}}{c_{Nd}+c_{Sb}}\right)}^{2}$$

where *η*_t1_ and *η*_t2_ refer to efficiency of ET channels from [ZrCl_6_]^2−^ octahedra to Nd^3+^ ions and from [SbCl_6_]^3−^ octahedra to Nd^3+^ ions, respectively. *R*_01_ and *R*_02_ are the critical distance of ET channels from [ZrCl_6_]^2−^ octahedra to Nd^3+^ ions and from [SbCl_6_]^3−^ octahedra to Nd^3+^ ions, respectively. *C*_Nd_, *C*_Zr_, and *C*_Sb_ correspond to the concentrations of Nd^3+^, Zr^4+^ and Sb^3+^. As a result, the value of *η*_t_/(1‐*η*_t_) is influenced by two factors: the critical distance *R*_0_ and the total concentration of sensitizer and activator. The critical distance *R*_0_ can be estimated by the following expression^[^ ^46^ ^]^:

(7) $$R_{0}^{6}=\frac{3h^{4}c_{light}^{4}Q_{A}}{4\pi n^{4}}\int_{0}^{\infty }\frac{f_{S}\left(E\right)F_{A}\left(E\right)}{E^{4}}dE$$

where *h* is the Planck constant, *c*_light_ is the speed of light, *f*_S_ is the normalized PL spectrum of sensitizer, *F*_A_ is the normalized absorption spectrum of activator, *n* is the refractive index of the host medium, *Q*_A_ is the oscillator strength of the absorption transition of the activator, and *E* is the average energy of the overlapping transition. For the two ET channels in Cs_2_ZrCl_6_:0.5%Sb^3+^,Nd^3+^, the critical distance *R*_0_ is proportional to the overlap integral of the normalized PL spectrum of sensitizer and the normalized absorption spectrum of activator. Considering the absorption peak of Nd^3+^ at 584 nm mentioned above, the PL spectrum of ^3^STEs possesses a larger overlap integral than that of the host STEs (Figure S5a, Supporting Information). As a result, the ET channel from the host STEs (ET1) shows a smaller *R*_0_ (*R*_01_ < *R*_02_) and a larger total concentration of sensitizer and activator. From the experimental results above, the ET1 possesses a larger ET efficiency *η*_t_, implying a larger value of *η*_t_/(1 − *η*_t_) (Figure S5b, Supporting Information). This result indicates that conmpared with ET2, the advantage of the sensitizer (Zr^4+^) concentration is enormously sufficient to overcome the disadvantage of the critical distance for ET1 to display larger *η*_t_ and *η*_t_/(1 − *η*_t_). Therefore, the sensitizer concentration plays the leading function in the comparison of the two ET efficiency. The possible PL mechanism is proposed in Figure 3d, for Cs_2_ZrCl_6_:Sb^3+^,Nd^3+^ MCs, after the excitation of 320 nm, the electrons in the ground‐state (^1^S_0_) are excited to the ^1^P_1_ and ^3^P_1_ excited‐states in [SbCl_6_]^3−^ octahedra along with the ET from ^1^P_1_ to ^3^P_1_, and then trapped to the singlet and triplet self‐trapped states, giving out cyan and orange broad emissions, corresponding to the recombination of ^1^STEs and ^3^STEs, respectively. At the same time, an ET process with a lower efficiency from triplet self‐trapped state to excited level of Nd^3+^ occurs. Besides, upon excitation at 256 nm, electrons are excited from the ground‐state to excited‐state in [ZrCl_6_]^2−^ octahedra, leading to blue emission resulting from the host STEs recombination. Meantime, partial energy is transferred from host self‐trapped state to excited level of Nd^3+^ with a higher efficiency. Finally, electrons located in the excited level of Nd^3+^ are relaxed to the ground‐state, resulting in the intense Nd^3+^ emission.

The PLQYs of Cs_2_ZrCl_6_, Cs_2_ZrCl_6_:0.5%Sb^3+^, and Cs_2_ZrCl_6_:0.5%Sb^3+^,(20–80%)Nd^3+^ MCs under the excitation of 256 and 320 nm are displayed in Table S4 (Supporting Information). Cs_2_ZrCl_6_:0.5%Sb^3+^,Nd^3+^ display decreasing PLQYs with the Nd^3+^ concentration increasing, which could be attributed to the increasing defect density caused by Nd^3+^ dopant. Furthermore, the stability of Cs_2_ZrCl_6_:0.5%Sb^3+^,80%Nd^3+^ MCs was investigated. As displayed in Figure S6a (Supporting Information), the thermogravimetric (TG) curve measured in nitrogen indicates that the perovskite MCs display no obvious weight loss (below 5%) up to 400 °C, indicating the remarkable structure stability of Cs_2_ZrCl_6_:0.5%Sb^3+^,80%Nd^3+^ MCs. In Figure S6b (Supporting Information), after being stored for 100 days, the MCs exhibit the essentially unchanged XRD pattern. Meantime, the PL emissions of the MCs could be remained at ≈90% of the initial intensities, manifesting the great air stability of Cs_2_ZrCl_6_:0.5%Sb^3+^,80%Nd^3+^ MCs (Figure S6c–f, Supporting Information).

**Figure 4:**
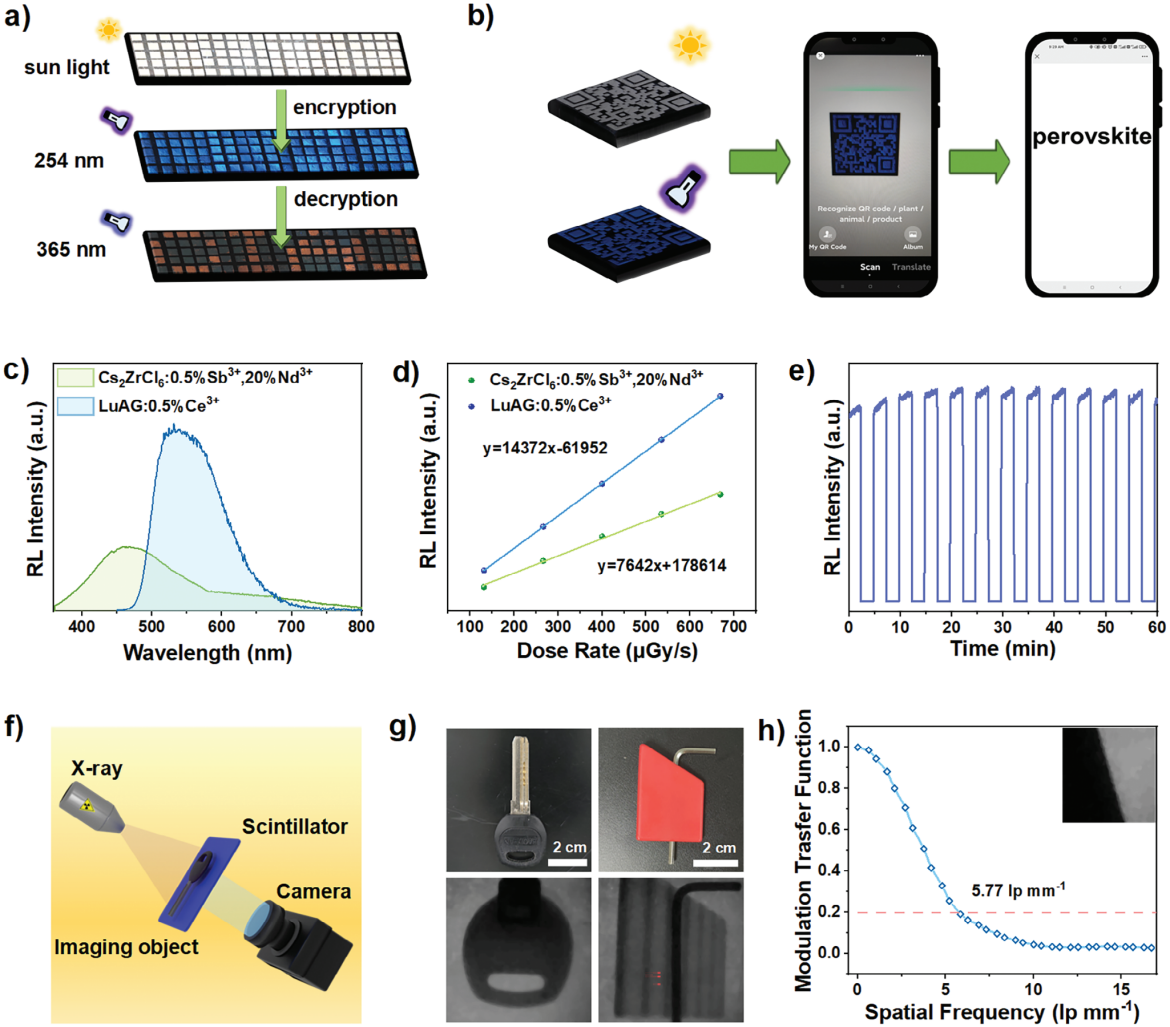
a) Images of security patterns made of pure Cs_2_ZrCl_6_ and Cs_2_ZrCl_6_:0.5%Sb^3+^,20%Nd^3+^. The decryption patterns emitting orange light upon excitation with 365 nm UV lamp were produced using Cs_2_ZrCl_6_:0.5%Sb^3+^,20%Nd^3+^, and the remaining area was filled with pure Cs_2_ZrCl_6_ to form encrypted patterns. b) Patterned QR code of Cs_2_ZrCl_6_:0.5%Sb^3+^,20%Nd^3+^ under visible light (top), and 254 nm UV light (bottom). Schematic diagram of as‐prepared QR code image is recognized directly by a commercial mobile phone application (WeChat) (right). c) RL spectra of Cs_2_ZrCl_6_:0.5%Sb^3+^,20% Nd^3+^, and LuAG:0.5%Ce^3+^ at dose rate of 669 µGy s^−1^. d) RL integral intensity of Cs_2_ZrCl_6_:0.5%Sb^3+^,20%Nd^3+^,^,^ and LuAG:0.5%Ce^3+^ as a function of X‐ray dose rate. e) RL stability of Cs_2_ZrCl_6_:0.5%Sb^3+^,20%Nd^3+^ under cyclical of X‐ray illumination at dose rate of 63 µGy s^−1^. f) Schematic of the X‐ray imaging system. g) The photographs of a metallic key and a metallic allen wrench under the visible light (top) and X‐ray (bottom) with X‐ray dose of 669 µGy s^−1^, which are all partially wrapped in plastic casing. h) MTF of the Cs_2_ZrCl_6_:0.5%Sb^3+^,20%Nd^3+^ thin film scintillation screen measured by the slanted‐edge method (inset).

Multi‐exciton emissive Cs_2_ZrCl_6_:0.5%Sb^3+^,Nd^3+^ MCs are not only suitable for exploring the mutiple ET processes, but also served as anti‐counterfeiting materials for dynamic color delivery in the visible region by tuning the excitation wavelength. Considering that excessive Nd^3+^ ions would quench the host STEs and dopant ^3^STEs emissions severely, Cs_2_ZrCl_6_:0.5%Sb^3+^,20%Nd^3+^ with the lowest Nd^3+^ concentration was selected among Cs_2_ZrCl_6_:0.5%Sb^3+^,(20‐80%)Nd^3+^ MCs. In Figure S7a–c (Supporting Information), it could be observed that Cs_2_ZrCl_6_:0.5%Sb^3+^,20%Nd^3+^ MCs render dynamic broad emissions from blue to orange with the excitation wavelength increasing from 250 to 380 nm, making it well suited for anti‐counterfeiting and information encryption. Undoped Cs_2_ZrCl_6_ and Cs_2_ZrCl_6_:0.5%Sb^3+^,20%Nd^3+^ powder were selectively filled into a pixelated pattern (5 × 20 dot matrix; **Figure** **4**a). These compounds show the same color under sun light (white) or 254 nm UV light (blue), and the encrypted message could not be decrypted at this moment. Then, the excitation light source was replaced by 365 nm UV light, the “CIAC” orange pattern composed of Cs_2_ZrCl_6_:0.5%Sb^3+^,20%Nd^3+^ powder could be observed to complete the decryption of encrypted information. Moreover, as shown in Figure 4b, by filling Cs_2_ZrCl_6_:0.5%Sb^3+^,20%Nd^3+^ powder into designed patterned grooves, delicate quick response (QR) code could be clearly presented. As‐prepared QR code image could be recognized directly by a commercial mobile phone application (WeChat), suggesting its prospects as high‐resolution PL imaging agent.

Apparently, the multiple STEs emissions of Cs_2_ZrCl_6_:0.5%Sb^3+^,20%Nd^3+^ MCs show large Stokes shifts, which is generally favourable for Cs_2_ZrCl_6_:0.5%Sb^3+^,20%Nd^3+^ MCs as the scintillator since self‐absorption caused by small Stokes shift could result in efficiency loss for scintillators.^[^ ^11^, ^48^ ^]^ In order to investigate the scintillation performance of Cs_2_ZrCl_6_:0.5%Sb^3+^,20%Nd^3+^, its absorption coefficient and X‐ray attenuation efficiency are compared with those of common scintillator Lu_3_Al_5_O_12_:0.5%Ce^3+^ (LuAG:0.5%Ce^3+^). In Figure S8a (Supporting Information), Cs_2_ZrCl_6_:0.5%Sb^3+^,20%Nd^3+^ MCs show lower absorption coefficient than LuAG:0.5%Ce^3+^ from 15 to 20 keV. Then, the samples with thickness of 0.8 mm (Cs_2_ZrCl_6_:0.5%Sb^3+^,20%Nd^3+^) and 1.0 mm (LuAG:0.5%Ce^3+^) were fabricated based on identical X‐ray attenuation efficiency (99%, at 17.5 keV) to unify the absorbed X‐ray energy of these two kinds of scintillators (Figure S8b, Supporting Information). X‐ray radioluminescence (RL) spectra of Cs_2_ZrCl_6_:0.5%Sb^3+^,20%Nd^3+^ MCs are presented in Figure 4c and Figure S8c (Supporting Information), host STEs emission and dopant ^3^STEs emission could be observed, which reveals the same luminescene mechanism as that excited by UV light. As the X‐ray dose rate increasing, the RL intensity of Cs_2_ZrCl_6_:0.5%Sb^3+^,20%Nd^3+^ increases and presents linear response curve, from which its X‐ray light yield (LY) could be obtained indirectly by means of the commercial scintillator LuAG:0.5%Ce^3+^ (the LY of 25000 photons MeV^−1^) as a reference (Figure 4d). Under the same X‐ray absorption cross sections, the response of Cs_2_ZrCl_6_:0.5%Sb^3+^,20%Nd^3+^ MCs is 0.532 times of LuAG:0.5%Ce^3+^ scintillator, thus the LY of Cs_2_ZrCl_6_:0.5%Sb^3+^,20%Nd^3+^ MCs is calculated as ≈13 300 photons MeV^−1^, which is close to that of Cs_2_ZrCl_6_:2.1%Sb^3+^ scintillator (18 000 ± 700 photons MeV^−1^) that reported recently.^[^ ^15^ ^]^ The lowest detection limit could determine the minimum dose rate required for detection and could be estimated when the signal‐to‐noise ratio (SNR) is 3. In Figure S8d (Supporting Information), the lowest detection limit of Cs_2_ZrCl_6_:0.5%Sb^3+^,20%Nd^3+^ MCs is estimated to be 1.424 μGy s^−1^, which is far lower than the typical medical X‐ray diagnostic requirement.^[^ ^49^ ^]^ Moreover, the RL intensity of the MCs exhibits negligible fluctuations under X‐ray cyclical irradiation for 1 h, implying the great radiation stability of Cs_2_ZrCl_6_:0.5%Sb^3+^,20%Nd^3+^ MCs (Figure 4e). To investigate the X‐ray imaging ability of Cs_2_ZrCl_6_:0.5%Sb^3+^,20%Nd^3+^ MCs, transparent and flexible scintillation film was fabricated by embedding the MCs powder into polydimethylsiloxane (PDMS) (5 cm × 5 cm × 0.1 cm; Figure S8e, Supporting Information). Subsequently, the object and flexible Cs_2_ZrCl_6_:0.5%Sb^3+^,20%Nd^3+^@PDMS thin film were put in self‐built X‐ray imaging system (Figure 4f). A metallic key and a metallic allen wrench partially wrapped in plastic casing were selected as the testing objects to verify X‐ray detection ability of Cs_2_ZrCl_6_:0.5%Sb^3+^,20%Nd^3+^@PDMS thin film. The X‐ray contrast images with distinct inside structure are successfully disclosed due to the difference X‐ray transmittance between metal and plastic (Figure 4g). The modulation transfer functions (MTF) of X‐ray images was calculated using slanted‐edge images to further evaluate image quality (Figure 4h).^[^ ^50^ ^]^ The spatial resolution of Cs_2_ZrCl_6_:0.5%Sb^3+^,20%Nd^3+^@PDMS thin film is acquired as 5.77 lp mm^−1^ at MTF = 0.2. Compared with X‐ray imaging of recently reported lead‐free perovskite scintillator Cs_2_ZnBr_4_:25%Mn@PDMS thin film (5.06 lp mm^−1^) and Cs_2_Ag_0.6_Na_0.4_In_0.85_Bi_0.15_Cl_6_ (4.4 lp mm^−1^), Cs_2_ZrCl_6_:0.5%Sb^3+^,20%Nd^3+^@PDMS thin film also exhibits great X‐ray imaging ability.^[^ ^11^, ^51^ ^]^ The above‐mentioned advantages of as‐designed Cs_2_ZrCl_6_:0.5%Sb^3+^,20%Nd^3+^ exhibit its potentiality as a candidate of X‐ray scintillator.

## 3 Conclusion

In summary, Sb^3+^/Nd^3+^ co‐doped Cs_2_ZrCl_6_ MCs with Nd^3+^ characteristic emission, host STEs emission and dopant singlet/triplet STEs emissions were prepared by a room temperature precipitation method. The multiple ET channels from the host STEs and dopant ^3^STEs to Nd^3+^ ions were explored and revealed. According to the Förster–Dexter ET theory, benefit from the high ion concentration of sensitizer rather than the small spectral overlap, the ET from the host STEs to Nd^3+^ ions exhibit the larger ET efficiency than that from the dopant ^3^STEs to Nd^3+^ ions. Sb^3+^/Nd^3+^ co‐doped Cs_2_ZrCl_6_ enables dynamic color delivery under selective excitation and exhibits great potential as multi‐mode anti‐counterfeiting material to encrypt multilevel optical codes. Furthermore, Sb^3+^/Nd^3+^ co‐doped Cs_2_ZrCl_6_ presents excellent X‐ray scintillation performance as well as promising X‐ray imaging ability. This work not only provides a new study perspective to understand multiple ET processes and the related influencing factors in rare earth doped multi‐exciton emissive perovskites, but also exhibits their great potentiality as anti‐counterfeiting materials and next generation X‐ray scintillators.

## Conflict of Interest

The authors declare no conflict of interest.

## Supporting information

Supporting Information

## Data Availability

The data that support the findings of this study are available from the corresponding author upon reasonable request.
