# Supplementary material for: Multiple Energy Transfer Channels in Rare Earth Doped Multi‐Exciton Emissive Perovskites
Source: Adv Sci (Weinh). 2023 Dec 21;11(9):2307354. doi: 10.1002/advs.202307354 (PMC10916588; doi:10.1002/advs.202307354)
Supplement: Supplementary file 1 — Supporting Information [file ADVS-11-2307354-s001.pdf]

ADVANCED SCIENCE
Open Access

Supporting Information

for *Adv. Sci.*, DOI 10.1002/advs.202307354

Multiple Energy Transfer Channels in Rare Earth Doped Multi-Exciton Emissive Perovskites

Huwei Li, Kai Han, Zheyu Li, Hongxia Yue, Xinyu Fu, Xinyu Wang, Zhiguo Xia, Shuyan Song, Jing Feng and Hongjie Zhang**

Supporting Information

Multiple Energy Transfer Channels in Rare Earth Doped Multi-Exciton Emissive Perovskites

Huwei Li, Kai Han, Zheyu Li, Hongxia Yue, Xinyu Fu, Xinyu Wang, Zhiguo Xia, Shuyan Song, Jing Feng, and Hongjie Zhang**

H. Li, Z. Li, H. Yue, X. Fu, X. Wang, S. Song, J. Feng, H. Zhang
State Key Laboratory of Rare Earth Resource Utilization
Changchun Institute of Applied Chemistry
Chinese Academy of Sciences
Changchun, Jilin 130022, China
E-mail: fengj@ciac.ac.cn; hongjie@ciac.ac.cn
H. Li, H. Zhang
State Key Laboratory of Inorganic Synthesis and Preparative Chemistry
College of Chemistry
Jilin University
Changchun, Jilin 130012, China
K. Han, Z. Xia
State Key Laboratory of Luminescent Materials and Devices
School of Physics and Optoelectronics
South China University of Technology
Guangzhou, Guangdong 510641, China
Z. Li, H. Yue, X. Fu, S. Song, J. Feng, H. Zhang
School of Applied Chemistry and Engineering
University of Science and Technology of China
Hefei, Anhui 230026, China
H. Zhang
Department of Chemistry
Tsinghua University
Beijing 100084, China

Methods

Chemicals: Cesium chloride (CsCl, 99.9%) and antimony trichloride ($SbCl_3$, 99.98%) were purchased from Aladdin. Zirconium tetrachloride ($ZrCl_4$, 99.5%) was purchased from Macklin. Neodymium chloride ($NdCl_3$, 99.9%) was purchased from Alfa Aesar. Methanol, ethanol and hydrochloric acid (HCl) were purchased from XiLONG SCIENCE. Polydimethylsiloxane (PDMS, SYLGARD® 184) was purchased from Dow Corning. All the chemicals were commercially purchased and used without further purification.

Synthesis of Cs_2ZrCl_6:Sb^{3+} and Cs_2ZrCl_6:Sb^{3+},Nd^{3+} microcrystals: For Cs_2ZrCl_6:0.5%Sb^{3+} MCs, 0.5 mmol CsCl was first dissolved in 1 mL of HCl under ultrasound. 0.25×(1-x) mmol $ZrCl_4$, and 0.25x mmol $SbCl_3$ (x = 0, 0.005, 0.01, 0.025, 0.05, 0.1) were dissolved in 1 mL HCl. Then, the two solutions were mixed evenly and a white precipitate was immediately generated. The product was extracted from the crude solution via centrifugation process (3000 rpm, 2 min), followed by washing with HCl solution first and then with EtOH. Finally, the white powder was dried at 60 °C to obtain perovskite MCs.

For Cs_2ZrCl_6:0.5%Sb^{3+},Nd^{3+} MCs, the similar synthesis process was adopted. 0.25y mmol $NdCl_3$ (y = 0, 0.2, 0.4, 0.6, 0.8) were dissolved in 1 mL of MeOH, and then this solution was added into the HCl mixed solution including 0.25×(0.995-y) mmol $ZrCl_4$ and 0.00125 mmol $SbCl_3$ along with being warm. The CsCl solution was quickly added into the above mixed solution before it restore to room temperature. The precipitated products are treated in the same way.

Synthesis of Cs_2ZrCl_6:0.5%Sb^{3+},20%Nd^{3+} flexible thin film: First, 2.5 g of PDMS base resin and 0.25 g of curing agent were mixed in a beaker. Then, 0.275 g of Cs_2ZrCl_6:0.5%Sb^{3+},20%Nd^{3+} powder was dispersed in the above PDMS precursor with stirring for 10 min. After curing at 100 ˚C for 60 min, the flexible composites were obtained.

Characterizations: The powder X-ray diffraction (XRD) patterns were recorded on a Bruker D8 Advance powder X-ray diffractometer using Cu Kα radiation (λ = 1.54 Å) and operating at a voltage of 40 kV and a current of 40 mA. The morphologies and element mapping were recorded by a field emission scanning electron microscope (FE-SEM, S-4800, Hitachi). The X-ray photoelectron spectroscopy (XPS) was conducted using a Thermo SCIENTIFIC ESCALAB 250Xi spectrometer. UV-visible absorption spectra were obtained using a Shimadzu 3600 UV-vis spectrophotometer. The PL spectra were recorded using a Horiba Jobin Yvon Fluorolog 3 fluorescence spectrometer equipped with a 450 W xenon lamp as the excitation source at room temperature. The temperature-dependent PL spectra were recorded using a fluorescence spectrometer equipped with a xenon lamp as the excitation source

(Edinburgh Instruments FLS-920) and a temperature controller (INSTEC temperature controller, American Instec). The fluorescent decay curves were obtained from a Lecroy Wave Runner 6100 Digital Oscilloscope (1 GHz) taking a tunable laser (pulse width = 4 ns, gate = 500 ns) and a μF900 lamp as the excitation sources. The element contents were measured using an inductively coupled plasma (ICP) optical emission spectrometer (ICAP6300, Thermo Scientific). The photoluminescence quantum yields (PLQYs) were obtained directly using an absolute PL quantum yield measurement system (C10028, Hamamatsu Photonics K. K., Japan). The corresponding RL spectra were recorded by FLS1000 fluorescence spectrophotometer (Edinburgh Instruments Ltd., U. K.) with an X-ray source (Amptek Mini-X tube with an Mo target and 6 W maximum power output). X-ray imaging were acquired by using a CMOS camera. Thermogravimetric analysis (TGA) spectrum was collected with the Perkin-Elmer STA 6000 instrument. The crystals are heated in the range 40-1000˚C at the heating rate of 10 ˚C per minute, under N^2 atmosphere. The measured PL decay curves were fitted with a mono-exponential decay function, as expressed: $I(t) = I_0 + Ae^{(-t/\tau)}$. A represents constant, τ is decay time for the exponential component, I_0 and $I(t)$ are PL intensities at time 0 and t.

Supporting Tables

Table S1. ICP-OES data of Cs_2ZrCl_6:Sb MCs.

Feeding ratios of Sb/(Sb+Zr)×100%	Sb/(Sb+Zr) ratios measured by ICP-OES
0.5%	0.68%
1%	0.73%
2.5%	1.70%
5%	2.44%
10%	3.95%

Table S2. ICP-OES data of Cs_2ZrCl_6:0.5%Sb^{3+},(20-80%)Nd^{3+} MCs.

Feeding ratio of Nd/(Zr+Sb+Nd)×100%	Nd/(Zr+Sb+Nd) ratios measured by ICP-OES	Sb/(Sb+Zr) ratios measured by ICP-OES
20%	1.46%	0.90%
40%	2.13%	0.72%
60%	3.15%	0.59%
80%	3.81%	0.71%

Table S3. The fitted PL lifetimes of Cs_2ZrCl_6:0.5%Sb^{3+},(0-80%)Nd^{3+} MCs monitored at 630 nm under the excitation of 320 nm.

Feeding ratios of Nd/(Zr+Sb+Nd)× 100%	PL lifetime τ_x (μs)
0%	9.22
20%	8.65
40%	8.61
60%	8.42
80%	7.93

Table S4. The PLQYs of Cs_2ZrCl_6, Cs_2ZrCl_6:0.5%Sb^{3+} and Cs_2ZrCl_6:0.5%Sb^{3+},80%Nd^{3+} MCs under the excitation of 256 nm and 320 nm.

Doping concentration / Excitation Wavelength	undoped	0.5%Sb	0.5%Sb, 20%Nd	0.5%Sb, 40%Nd	0.5%Sb, 60%Nd	0.5%Sb, 80%Nd
256 nm	52.3%	40.8%	31.5%	26.2%	24.5%	21.7%
320 nm	N.A.	38.4%	32.8%	31.9%	30.2%	25.0%

Supporting Figures

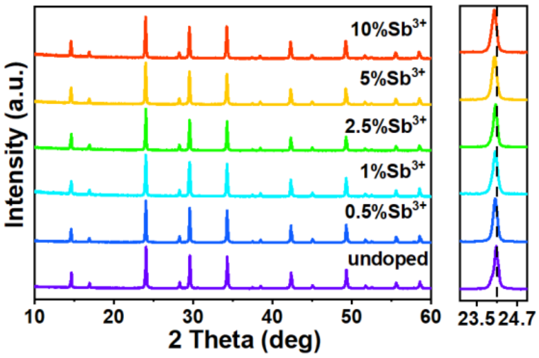

Figure S1. Powder XRD patterns (left panel) and magnified XRD peaks (right panel) of Cs_2ZrCl_6:Sb^{3+} MCs.

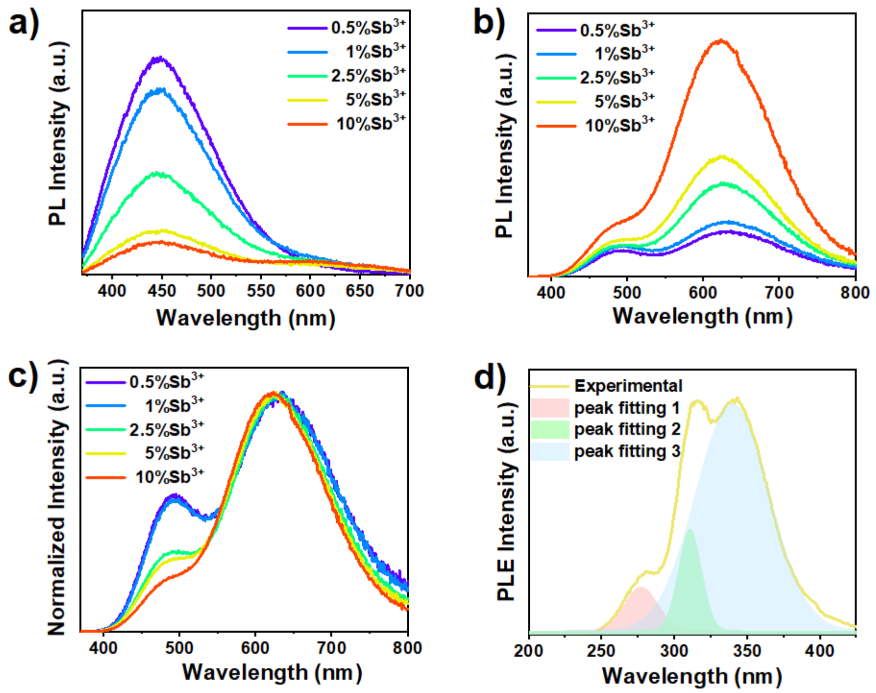

Figure S2. PL spectra of Cs_2ZrCl_6:(0.5-10%)Sb^{3+} MCs in visible region excited by a) 256 nm and b) 320 nm. c) Normalized PL spectra of Cs_2ZrCl_6:(0.5-10%)Sb^{3+} MCs in visible region excited by 320 nm. d) PLE spectrum of Cs_2ZrCl_6:0.5%Sb^{3+} monitored at 630 nm.

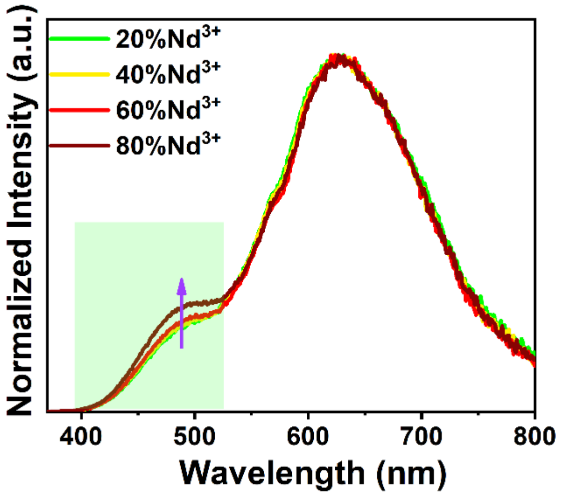

Figure S3. Normalized PL spectra of Cs_2ZrCl_6:0.5%Sb^{3+},(20-80%)Nd^{3+} MCs in visible region excited by 320 nm.

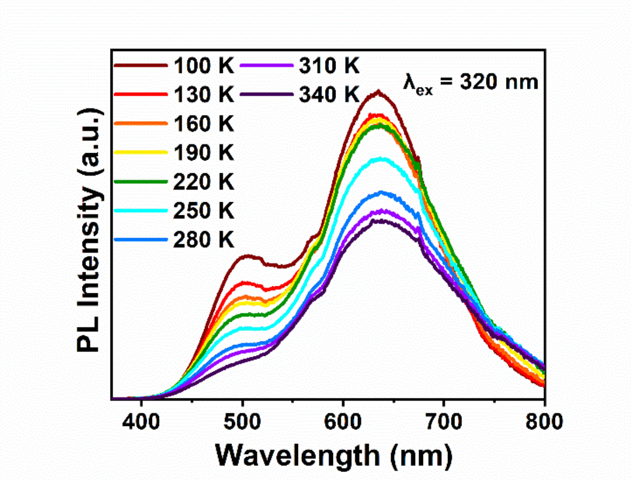

Figure S4. PL spectra versus temperature of Cs_2ZrCl_6:0.5%Sb^{3+},80%Nd^{3+} MCs in visible region excited by 320 nm.

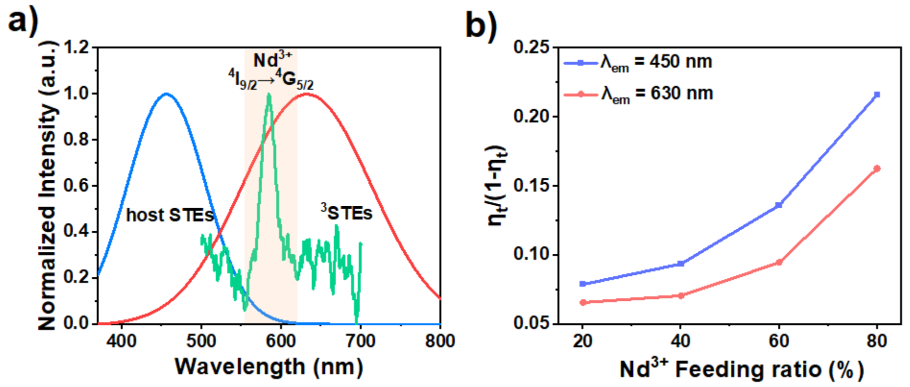

Figure S5. a) The normalized PL spectra of host STEs excited by 256 nm (blue) and dopant ^{3}STEs excited by 320 nm (red), and the normalized absorption spectrum of Nd^{3+} ions in Cs_2ZrCl_6:0.5%Sb^{3+},80%Nd^{3+} MCs (green). b) The $\eta_t/(1-\eta_t)$ variation of Cs_2ZrCl_6:0.5%Sb^{3+},(20-80%)Nd^{3+} MCs (monitored at 450 nm and 630 nm) as a function of Nd^{3+} feeding ratio.

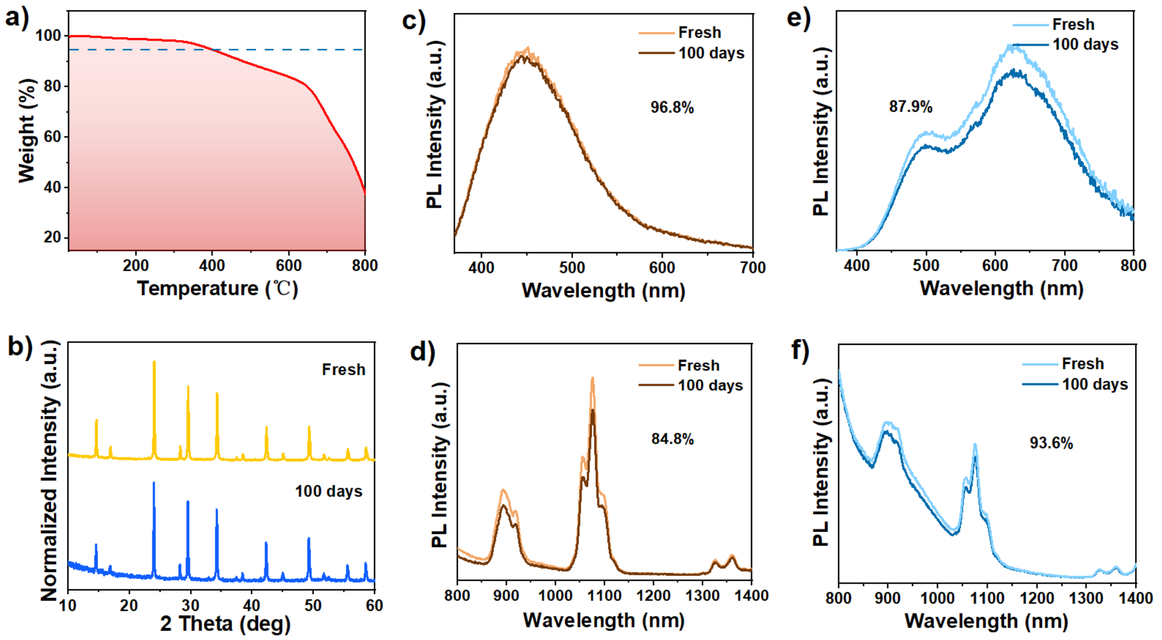

Figure S6. a) TG curve of Cs_2ZrCl_6:0.5%Sb^{3+},80%Nd^{3+} MCs. b) XRD patterns of fresh Cs_2ZrCl_6:0.5% Sb^{3+},80%Nd^{3+} and Cs_2ZrCl_6:0.5%Sb^{3+},80%Nd^{3+} stored in air for 100 days. PL spectra of fresh Cs_2ZrCl_6:0.5%Sb^{3+},80%Nd^{3+} and Cs_2ZrCl_6:0.5%Sb^{3+},80%Nd^{3+} stored in air for 100 days in visible region excited by c) 256 nm and e) 320 nm. PL spectra of fresh Cs_2ZrCl_6:0.5%Sb^{3+},80%Nd^{3+} and Cs_2ZrCl_6:0.5%Sb^{3+},80%Nd^{3+} stored in air for 100 days in NIR region excited by d) 256 nm and f) 320 nm.

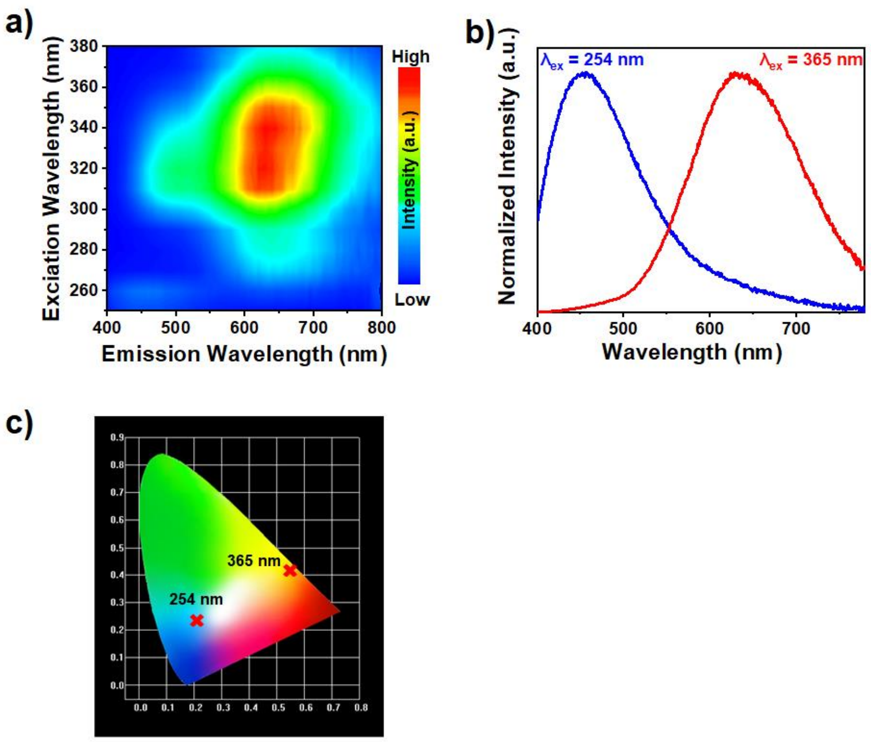

Figure S7. a) Excitation–emission color map of Cs_2ZrCl_6:0.5%Sb^{3+},20%Nd^{3+} MCs. b) Normalized PL spectra of Cs_2ZrCl_6:0.5%Sb^{3+},20%Nd^{3+} MCs excited by 254 nm and 365 nm. c) The CIE coordinates of Cs_2ZrCl_6:0.5%Sb^{3+},20%Nd^{3+} MCs under different excitation wavelength plotted on the CIE 1931 chromaticity chart.

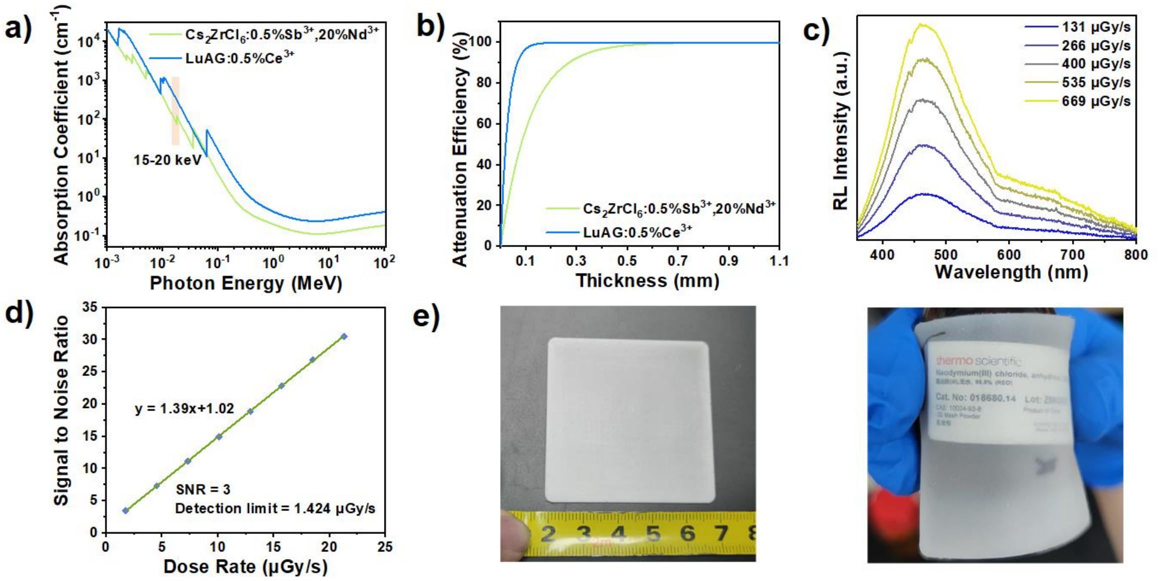

Figure S8. a) The absorption coefficients of Cs_2ZrCl_6:0.5%Sb^{3+},20%Nd^{3+} MCs and LuAG:0.5%Ce as a function of photon energy from 10^{-3} MeV to 10^{2} MeV. b) X-ray attenuation efficiency of Cs_2ZrCl_6:0.5%Sb^{3+},20%Nd^{3+} MCs and the typical scintillator LuAG:0.5%Ce as a function of the thickness (X-ray photon energy of 17.5 keV). c) RL spectra of Cs_2ZrCl_6:0.5%Sb^{3+},20%Nd^{3+} MCs versus X-ray dose rate. d) Linear relationship between signal to noise ratio value and X-ray dose rate. e) Photographs of large-sized (left) and transparent (right) Cs_2ZrCl_6:0.5%Sb^{3+},20%Nd^{3+}@PDSM thin film under visible light.
